# Retrospective evaluation of surgical outcomes using traditional, internal obturator muscle flap, and sacroischial sling technique for canine perineal hernia repair

**DOI:** 10.5455/javar.2025.l1002

**Published:** 2025-12-30

**Authors:** Wijit Sutthiprapa, Pharkpoom Budsayaplakorn, Nattika Koatsang, Wutthiwong Theerapan, Naris Thengchaisri

**Affiliations:** 1 Surgery Unit, Kasetsart University Veterinary Teaching Hospital, Bangkok, Thailand; 2 Department of Companion Animal Clinical Sciences, Faculty of Veterinary Medicine, Kasetsart University, Bangkok, Thailand

**Keywords:** Dogs, IOMF, leader line, perineal hernia, surgical complication

## Abstract

**Objectives:**

This retrospective study compared the outcomes of three surgical methods: traditional technique, internal obturator muscle flap, and sacroischial sling (TT, IOMF, and SS) for the treatment of canine perineal hernias. Postoperative complications associated with each technique were also compared.

**Materials and Methods:**

87 dogs (86 males, 1 female) with perineal hernia were included in this study. Dogs were grouped based on the surgical technique used: TT (30 sites in 24 dogs), IOMF (30 sites in 26 dogs), and SS (53 sites in 37 dogs).

**Results:**

Surgical times were 36.8 ± 9.7 min for TT, 50.2 ± 13.6 min for IOMF, and 31.9 ± 11.53 min for SS. Both TT and SS were significantly faster than IOMF (*p* < 0.01). A comparative analysis of surgical outcomes revealed differing success and failure rates. The success rate of the IOMF group was higher (99.3%) compared to the TT group (80%); however, this difference was not statistically significant (*p* = 0.254). In contrast, the SS group demonstrated a statistically significantly greater success rate (98.1%) than the TT group (*p* = 0.008), indicating that it may be a more successful approach for perineal hernia correction in dogs. The TT group had the highest rate of temporary stranguria (20.8%) and required colopexy and cystopexy most frequently (16.7%). The SS group had the lowest rate of urinary incontinence (2.7%) and external anal sphincter muscle paresis (2.7%). However, this group exhibited the highest incidence of temporary dyschezia (8.1%) and a slightly elevated incidence of skin dehiscence. Wound complications were similar across all groups. The IOMF group had a higher incidence of external anal sphincter muscle paresis (26.9%) compared to both TT and SS. The complication rate of the SS group (7.0%, 13/185 events) was significantly lower than both TT (18.3%, 22/120; *p* < 0.01) and IOMF (18.5%, 24/130; *p* < 0.01) groups and required fewer additional procedures, indicating fewer overall complications.

**Conclusion:**

Overall, the SS technique is a practical, low-complication alternative for perineal hernia correction, offering results comparable to those of IOMF and superior to those of TT.

## Introduction

A perineal hernia results from the disruption of pelvic supportive structures, leading to the weakening of the pelvic diaphragm, often involving the levator ani muscle [1–3]. This disorder causes displacement of abdominal organs into the perineal area, resulting in perineal swelling[4]. Several theories have implicated prostatomegaly, chronic constipation, and hormonal changes in perineal hernia development; however, the exact etiology remains unclear [2,3,5]. Contributing factors include tenesmus, gender-related variations in muscle anatomy, hormonal influences, nerve-related muscle atrophy [2,5–7], complications of radical pelvic oncologic procedures for recto-anal cancer [8], as well as prostate hyperplasia, myopathy, and upregulation of relaxin receptors in pelvic muscles. While a perineal hernia most often affects intact male dogs, it can also occur in females [7]. The four main types of perineal hernia are defined based on the affected anatomical structures: [1]caudal hernia, involving the levator ani muscle, internal obturator, and external anal sphincter muscle;[2] dorsal hernia, affecting the coccygeus and levator ani muscle;[3] sciatic hernia, involving the coccygeus muscle and sacrotuberous ligament; and [4]ventral hernia, affecting the ischiourethralis, bulbocavernosus, and ischiocavernosus muscles [3,5]. Surgical correction typically involves pelvic diaphragm reconstruction using techniques including the traditional technique (TT) with imbrication [3], internal obturator muscle flap (IOMF) [9,10], semitendinosus muscle transposition [11], autogenous fascia lata graft [12], tunica vaginalis [13,14], synthetic mesh [15,16], and abdominal organopexy [17]. These methods vary in success rates and have distinct advantages and limitations in clinical practice.

For chronic perineal hernias with muscular atrophy, the sacrotuberous ligament can be used for lateral repair. Some studies recommend suturing through the ligament rather than around it to avoid entangling vessels and nerves [18]. To avoid sciatic nerve entrapment, a modified double purse-string technique for internal obturator muscle transposition herniorrhaphy has been developed [19]. In this technique, both the sacrotuberous ligament and internal obturator muscle are secured with two sutures to facilitate the closure of the pelvic diaphragm. However, in severe chronic hernias, concurrent abdominal organopexy has been effective in reducing recurrence [17,20]. The sacroischial sling (SS) technique is a useful surgical approach for managing perineal hernia in dogs with significant muscle atrophy. Nonetheless, its effectiveness in preventing recurrence of perineal hernia and minimizing complications, such as sciatic nerve entrapment, has not yet been reported.

The objectives of this study were to retrospectively compare the postoperative outcomes of three surgical techniques for perineal hernia repair in dogs: the TT, the IOMF, and the SS. Surgical time and hernia recurrence were compared among the three surgical methods. Additionally, the association between the surgical method and the frequency of postoperative complications was also evaluated.

## Materials and Methods

### Ethical approval

All animal studies were ethically reviewed and conducted in accordance with the guidelines and regulations of the Ethics of Animal Experimentation of the National Research Council of Thailand. The procedures in this study were approved by the Kasetsart University Institutional Animal Care and Use Committee (ACKU67-VET-116).

### Study period and location

A retrospective study was conducted at Kasetsart University Veterinary Teaching Hospital between May 2013 and August 2015. A total of 87 dogs exhibiting perineal hernias (113 affected sites) and presenting perineal swelling for the first time were included.

### Animals

A detailed comparison of perineal hernias treated with three surgical techniques (TT, IOMF, and SS groups) is shown in Table 1. The TT group consists of 24 dogs, with Pomeranians [6], Poodles [5], Chihuahuas [5], Shih Tzus [2], Pugs [1], French Bulldogs [1], and Mixed breeds [4]. The IOMF group includes 26 dogs, with Pomeranians [3], Poodles [4], Chihuahuas [3], Siberian Huskies [8], and Mixed breeds [8]. The SS group consists of 37 dogs, with Shih Tzus [7], Pomeranians [4], Poodles [3], Chihuahuas [3], Pugs [4], French Bulldogs [3], Siberian Huskies [5], and Mixed breeds [8]. Dogs in each surgical group were further classified into three weight categories: Small (S = 1–10 kg), Medium (M = 10.1–25 kg), and Large (L = over 25 kg). The distribution of unilateral and bilateral hernias was different in each group. The TT procedure was performed in 75.0% of unilateral hernias and 25.0% of bilateral hernias. The IOMF technique was applied in 84.6% of unilateral and 15.4% of bilateral cases, while the SS technique was used in 56.8% of unilateral and 43.2% of bilateral cases. The duration of each surgical procedure, as well as any surgical complications, was also recorded.

**Table 1.** General characteristics of dogs undergoing surgical correction for perineal hernia using three different surgical techniques.

Parameters	TT	IOMF	SS
Number of dogs	24	26	37
Unilateral hernia	18 (75.0%)	22 (84.6%)	21 (56.8%)
Bilateral hernia	6 (25.0%)	4 (15.4%)	16 (43.2%)
Age (years)	9.8 ± 2.8	8.8 ± 2.7	9.2 ± 2.3
Body weight (kg)	12.5 ± 8.52	11.6 ± 6.8	10.7 ± 7.22
Body weight category			
Small (≤10 kg)	12 (50.0%)	14 (53.8%)	33 (89.2%)
Medium (10.1–25 kg)	10 (41.7%)	9 (34.6%)	3 (8.1%)
Large (>25 kg)	2 (8.3%)	3 (11.5%)	1 (2.7%)
Sex			
Male	24 (100.0%)	26 (100.0%)	35 (94.6%)
Female	0 (0.0%)	0 (0.0%)	2 (5.4%)
Duration of symptoms prior to surgery			
> 1 month	20 (83.3%)	18 (69.2%)	26 (70.3%)
≤ 1 month	4 (16.7%)	8 (30.8%)	11 (29.7%)
Surgical time (min)	36.8 ± 9.7	50.2 ± 13.6^**^	31.9 ± 11.53 ^##^

^**^p < 0.01 versus TT, ^##^*p* < 0.01 versus IOMF.

### Anesthesia

Premedication was initiated with diazepam (Government Pharmaceutical Organization, Thailand) at a dosage of 0.3 mg/kg administered intravenously (IV). Anesthesia induction utilized propofol (Troypofol, Troikaa Pharmaceuticals, India) with a dose range of 2 to 4 mg/kg i.v., followed by endotracheal intubation. Anesthesia maintenance was achieved using isoflurane (Attane TM, Piramal Critical Care, Inc., USA) at a concentration of 1.5% to 2.0% in 100% oxygen. Prophylactic administration of antibiotics was performed with cefazolin (Cefaben, L.B.S. Laboratory Ltd., Thailand) at a dose of 20 mg/kg administered IV. Analgesia was provided through morphine (M & H Manufacturing, Company Ltd. For Food and Drug Administration, Thailand) at a dose of 0.5 mg/kg, supplemented by epidural administration of bupivacaine (Marcaine 0.5%, AstraZeneca AB, Sodertalje, Sweden) at a dose of 1 mg/kg, mixed with morphine (M & H Manufacturing, Company Ltd. For Food and Drug Administration, Thailand) at a dose of 0.1 mg/kg. Postoperative analgesia was administered using carprofen (Rimadyl, Inovat Industria Farmaceutica Ltd, Brazil) at a subcutaneous dose of 2.2 mg/kg.

### Surgical procedures

Hair was clipped from the caudal part of the body to the mid-tail in long-tailed dogs. Preoperatively, urinary bladder catheterization was performed to facilitate urine drainage and identify the urethra. Anal sacs were manually expressed, and the rectum was emptied of feces. A povidone-iodine-soaked gauze was then inserted into the rectum. Patients were positioned in sternal recumbency on the surgical table, with their tail fixed over their back, pelvis, and hindquarters elevated, and their hind legs padded, allowing them to hang down. A purse-string suture of 3/0 nylon was placed around the anal orifice. Aseptic preparation consisted of an alcohol-based surgical scrub using chlorhexidine gluconate 4% (Hexene, Osoth Inter Laboratory Company Ltd., Thailand) and isopropyl alcohol. Dogs were continually monitored throughout the surgical procedure and the recovery period for changes to the electrocardiogram, heart rate, respiratory rate, body temperature, oxygen saturation, and blood pressure.

**Figure 1.**
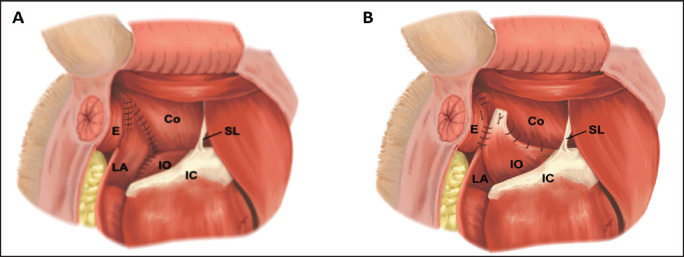
Surgical procedures for perineal hernia repairs in dogs, showing key anatomical details and suture placements. A: TT used in this study. Sutures (black lines) are placed between the external anal sphincter (E) or levator ani (LA) and the coccygeus (Co) muscle laterally, and between the external anal sphincter (E) or levator ani (LA) and the internal obturator (IO) muscle ventromedially. The sacrotuberous ligament (SL) and ischial arch (IC) serve as key anchoring points. B: IOMF. In addition to the suture placements described in A, this method includes extra sutures between the coccygeus (Co) and IO muscles, sacrotuberous ligament (SL), and ischial arch (IC) ventrolaterally, providing enhanced muscular support.

A dorsoventral incision was made from the base of the tail to the ischial arch. Surgical techniques, including TT (Fig. 1A) and IOMF (Fig. 1B), were performed. The most commonly herniated contents were retroperitoneal fat, omentum, and an enlarged prostate gland. The urinary bladder and small intestine were less frequently found. Herniated organs were carefully repositioned, and surrounding structures, such as the perineal muscles, rectum, and anal sacs, were examined. In cases with minor rectal diverticulum, a two-layer closure was performed using monofilament absorbable sutures (PDS 2-0, PDS II, Ethicon, UK). The SS technique is shown in Table 2andFig. 2,Fig. 3.Loose tissue over the ischial arch was removed to clearly identify the internal pudendal artery, vein, and nerve. Two holes were drilled through each ischial bone (using a 0.15–0.3 mm bit), one lateral and one medial, about 0.5 cm from the bone’s caudal edge, covering two-thirds of the ischial floor. The leader line was passed dorsoventrally through the cranial space of the fourth sacrum. Once positioned, the needle was removed, and the line was secured with a crimp clamp below the rectum, at least 1 cm from the ischial bone (Fig. 3). The leader line was then passed through each medial and lateral hole and tied twice using a surgeon’s knot, ventral to the ischium. Follow-up suture imbrication was performed using non-absorbable monofilament sutures (2-0 nylon or polypropylene). Sutures were pre-placed at 1 cm intervals between the leader line, external anal sphincter, and levator ani muscle, but not tied immediately. Ventral suturing began along the midline at the external anal sphincter. The lateral sutures were then tied to close the hernia gap. Hernia closure was confirmed by digital palpation. Standard closure procedures were employed for the subcutaneous tissue, utilizing a simple interrupted pattern with monofilament absorbable suture 2-0 (PDS II, Ethicon, UK). The skin was closed using monofilament polyamide suture 3-0 (Dafilon, B. Braun Melsungen, Germany).

**Figure 2.**
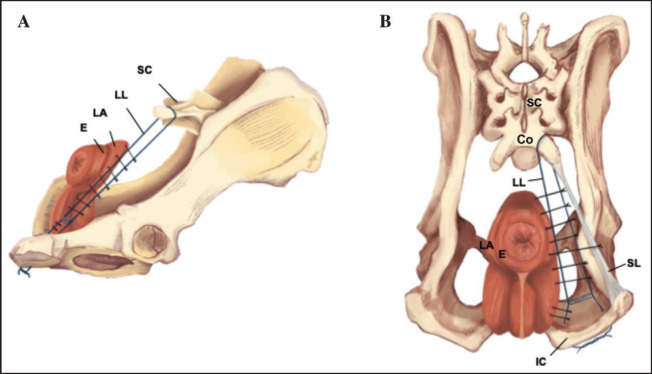
Perineal hernia repair using the SS technique, highlighting suture placement and key anatomical structures. A: Lateral view. B: Caudal view. Sutures are spaced 1–2 cm apart and placed between the external anal sphincter (E) or levator ani (LA) muscles and the polyethylene leader line (LL), both laterally and medially. Labeled structures include the sacrum (SC), leader line (LL), external anal sphincter (E), levator ani (LA), sacrotuberous ligament (SL), and ischial arch (IC), illustrating the configuration of the sling.

**Figure 3.**
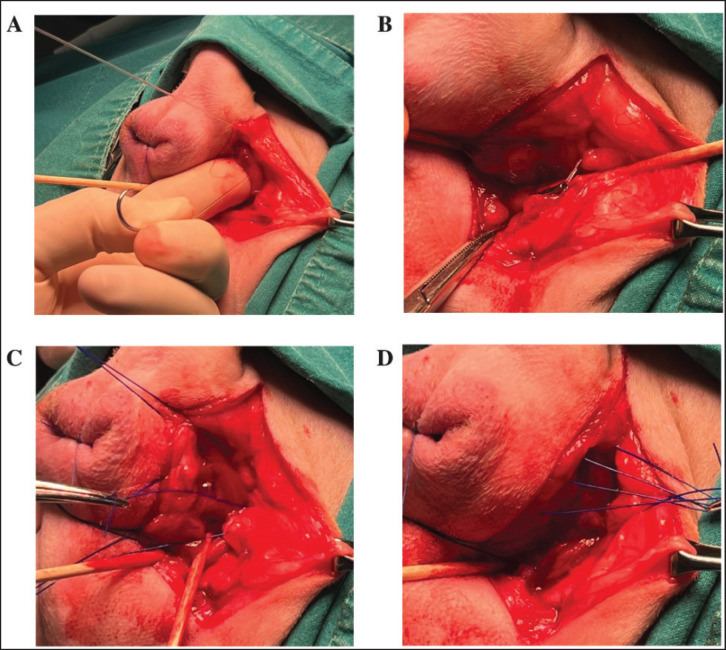
Step-by-step procedure of the SS technique for perineal hernia repair. A: The leader line (LL) is passed through the lateral inter-transverse process of the sacrum. B: It is then securely crimped about 1 cm above the sacral floor, with crimp tubes positioned beneath to anchor the sling. C: Monofilament non-absorbable <url>(2-0)</url> sutures are placed between the external anal sphincter or levator ani muscle and the lateral sections of the leader line. D: Tension is applied to the sutures before final tying to close the hernia effectively. Key structures such as the LL, crimp tubes, and muscle attachments are labeled for clarity.

**Figure 4.**
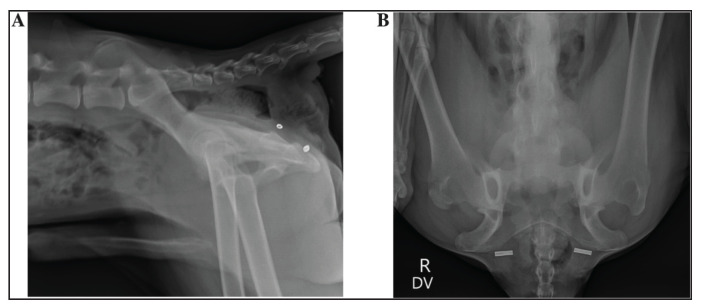
Radiographs 3 months after bilateral perineal hernia repair using the SS technique. A: Lateral view showing both crimp tubes positioned about 1 cm above the ischial floor. B: Dorsoventral view showing the crimp tubes centered near the ischial floor on each side. Arrows indicate the exact location of the crimp tubes used to secure the leader line.

All intact male dogs with muscle atrophy and weakness associated with canine prostatic hyperplasia underwent castration. Post-operative opiate analgesics (as above) were administered as needed, together with gabapentin (10 mg/kg) every 8 h for 5 days. Carprofen (Rimadyl, Inovat Industria Farmaceutica Ltd, Brazil) was used for postoperative analgesia at a dose of 4.4 mg/kg SC every 24 h for 4 days. Oral cephalexin (22 mg/kg per dose) was administered twice daily for 7–10 days postoperatively. Owners were contacted on day 4 to report any potential postoperative wound complications. A digital rectal examination was conducted on day 7. To avoid constipation, a stool softener was prescribed. A follow-up appointment at the clinic was scheduled to address any underlying issues. The clinic also contacted owners on postoperative days 30, 60, and 90 to assess complications. This evaluation included an assessment of urinary, digestive, wound, and neuromuscular disorders, as well as the need for colopexy or cystopexy. This comprehensive approach ensured thorough monitoring until complete recovery was achieved. A radiographic evaluation was also performed postoperatively (Fig. 4).

### Statistical analysis

Statistical analysis of canines was conducted using STATA 12 (StataCorp, College Station, TX). Data are presented as percentages, and Fisher’s exact test was employed to examine the association between the number of failures (recurrence of perineal hernia within 3 months) and the surgical technique used for perineal hernia correction. The proportion of dogs with postoperative complications such as urinary, digestive, wound, and neuromuscular disorders, or the need for colopexy, cystopexy, or vas deferentopexy, was also compared using binomial statistics to assess the effectiveness of the treatments. A *p*-value of less than 0.05 was considered statistically significant.

### Results

The timing of surgical interventions varied among treatment groups. The TT procedure was primarily used for cases lasting more than 1 month (83.3%), with only 16.7% of cases presenting for less than 1 month. The IOMF technique was applied to 69.2% of cases lasting longer than 1 month and to 30.8% of cases lasting less than 1 month. Similarly, the SS method was predominantly used for cases exceeding 1 month (70.3%), with 29.7% of cases presenting within 1 month. Minor differences were observed in the age and body weight of dogs. The TT group had an average age of 9.8 years and a mean body weight of 12.5 ± 8.52 kg. The IOMF group had an average age of 8.8 ± 2.7 years and a mean body weight of 11.6 ± 6.8 kg. The SS group had an average age of 9.2 ± 2.3 years and a mean body weight of 10.7 ± 7.22 kg.

All dogs recovered uneventfully from the surgery for a perineal hernia. Surgical times varied: the TT group averaged 36.8 ± 9.7 min, the IOMF group required 50.2 ± 13.6 min, and the SS group had the shortest mean surgical time of 31.9 ± 11.53 min (Table 1). Completion of both TT and SS procedures was significantly faster than IOMF (*p* < 0.01).

The surgical success and failure rates for perineal hernia correction in dogs are shown in Table 3. The TT group had an 80% success rate (24/30) and a 20% failure rate (6/30). The IOMF group had a higher success rate of 93% (28/30) and a 7% failure rate (2/30), but the difference compared to TT was not statistically significant (*p* = 0.254). The SS group exhibited a 98% success rate (52/53) and a 2% failure rate (1/53), which was a statistically significant improvement over the TT group (*p* = 0.008). There was no statistically significant difference between the IOMF and SS groups.

A comparative analysis of postoperative complications is presented in Table 4. For urinary disorders, the TT group had the highest rate of temporary stranguria (20.8%), while the SS group had the lowest rate of urinary incontinence (2.7%). Among digestive complications, temporary dyschezia was most common in the SS group (8.1%). Wound complications were similar across all groups, although the SS group had a slightly higher incidence of skin dehiscence. External anal sphincter muscle paresis was less frequent in the SS group (2.7%) compared to the TT (20.8%) and IOMF (26.9%) groups. The difference between the SS and TT groups was statistically significant (*p* < 0.05). Additionally, the TT group required colopexy and cystopexy more frequently (16.7%) than the IOMF and SS groups. The SS group had the lowest overall complication rate (7.0%, 13/185 events), which was significantly lower compared to both the TT group (18.3%, 22/120 events; *p* < 0.01) and the IOMF group (18.5%, 24/130 events; *p* < 0.01).

**Table 2.** Characteristics of the leader line chosen in dogs undergoing perineal hernia surgery with the SS technique.

Sizes of leader line	Diameter of nylon	Size of dogs
50 lb	0.7 mm.	<10.0 kg.
80 lb	0.9 mm.	10.1–25.0 kg.
100 lb	1.0 mm.	>25.0 kg

**Table 3.** Comparison of surgical success and failure rates for perineal hernia in dogs undergoing three different procedures: TT, IOMF, and SS techniques.

Surgical techniques	Surgical sites	Number of success (%)	Number of failures (%)
TT	30	24 (80%)	6 (20.0%)
IOMF	30	28 (99.3%)	2 (6%)
SS technique	53	52 (98.1%)	1 (1.8%)^ **^

^**^p < 0.01 versus TT.

**Table 4.** Post-operative complications following perineal hernia surgery: a comparison of three different surgical techniques.

Complication category (*N* = number of dogs)	TT ( *N* = 24)	IOMF ( *N* = 26)	SS ( *N* = 37)
Urinary disorders			
Temporary stranguria	5 (20.8%)	3 (11.5%)	4 (10.8%)
UTI	–	–	–
Urinary incontinence	2 (8.3%)	1 (3.8%)	1 (2.7%)
Digestive disorders			
Temporary dyschezia	2 (8.3%)	2 (7.7%)	3 (8.1%)
Hematochezia	–	–	–
Rectal leakage (abscess)	1 (4.2%)	–	–
Wound complications			
Seroma	–	3 (11.5%)	–
Infection	–	2 (7.7%)	1 (2.7%)
Partial skin dehiscence	2 (8.3%)	4 (15.4%)	2 (5.4%)
Complete skin dehiscence	–	–	1 (2.7%)
Neuromuscular disorders			
Sciatic nerve entrapment	1 (4.2%)	–	–
External anal muscle paresis	5 (20.8%)	7 (26.9%)	1 (2.7%) ^*##^
Required colopexy/cystopexy	4 (16.7%)	2 (7.7%)	–
Overall events of complications	22/120 (18.3%)	24/130 (18.5%)	13/185 (7.0%) ^**##^

^*^p < 0.05 versus TT, ^**^p <0.01 versus TT, ^##^*p* < 0.01 versus IOMF.

### Discussion

The study compared the breed, age, and sex characteristics of affected dogs to those documented in previous studies on populations with perineal hernia [21–23]. The most commonly affected breeds were mixed, Siberian huskies, and Shih Tzus. The average age of affected dogs (7 to 9 years) was consistent with previous reports. As previously reported, intact male dogs were more frequently affected by perineal hernia than female dogs [24]. This predisposition in intact males is likely due to the weakening of pelvic diaphragm muscles, possibly influenced by relaxin [6,25]. Relaxin is present in hypertrophic prostates[26] and may contribute to the weakening of the pelvic diaphragm muscle and connective tissue during hernia formation. This muscle weakness can result in visible unilateral or bilateral abnormalities, as pelvic or abdominal organs protrude into the subcutaneous perineal region. While most common in male dogs, perineal hernias can occur in female dogs and are often associated with specific uterine pathologies [7,27].

The SS is a promising surgical procedure, which is reflected in its shorter surgical time compared to the IOMF technique and similar to the TT procedure. The success rate in the SS group was 98% (52/53), with a 2% failure rate (1/53), which was not significantly different from the IOMF group. Interestingly, the IOMF procedure, traditionally considered the gold standard for treating perineal hernias with a success rate exceeding 90% [4], did not demonstrate a significant advantage over the SS technique in the present study. While our study’s retrospective nature limits control over all variables, the SS technique demonstrated lower recurrence and complication rates compared to the TT and IOMF methods, suggesting its effectiveness even in complex or chronic cases. Surgical skill and proper suturing are critical in muscle transposition procedures to prevent recurrence [23–28]. As noted in a previous study, surgeon experience can influence outcomes, with good results achievable from various methods [4]. For complex cases such as bilateral or recurrent perineal hernia, combined techniques including bilateral herniorrhaphy with colopexy, cystopexy, or bilateral deferentopexy have resulted in 93% of dogs remaining free of clinical signs. A two-step laparotomy procedure has also shown high success in recurrent cases. In contrast, internal obturator muscle transposition has been associated with a 27.4% recurrence rate, with postoperative tenesmus identified as a potential risk factor [21–23].

In this study, the TT group had a higher failure rate (20%) compared to the IOMF (6%) and SS (1.8%) groups. The difference between TT and SS was statistically significant (*p* = 0.008). While hormonal imbalances may contribute to muscle atrophy and hernia formation [26], our findings suggest that recurrence in perineal hernia cases may be more strongly influenced by the surgical technique, choice of suture materials, or the inherent weakness in the pelvic diaphragm muscle structure [29]. Synthetic materials, such as polyamide and catgut, have been shown to reduce recurrence rates compared to biological materials [29]. In this study, the TT procedure was selected in some cases by owners based on financial considerations and client education. In contrast, the IOMF technique was performed after careful identification of key pelvic diaphragm muscles. The TT procedure achieved acceptable success rates; however, the SS technique provided superior overall outcomes, with a significantly lower complication rate (7.0%) compared to both the TT (18.3%) and IOMF (18.5%) methods (*p* < 0.01).

The SS technique was also employed in this study to treat recurrent hernias, even when identifying the three main muscles was challenging due to the patient’s condition. Surgical approaches for treating perineal hernias are continually refined to achieve favorable post-surgical outcomes with cost-effective and straightforward procedures. The goal of each technique is to provide adequate strength and stability to prevent hernia recurrence while minimizing the risk of infection in the surgical wound area. Failed surgical interventions were often reported to originate at the ventral aspect of the surgical site, with success largely dependent on the condition of the surrounding muscle tissue. In some cases, particularly in smaller dogs or those with prolonged herniation, the muscle flap used in IOMF lacked sufficient strength or bulk to allow closure of the herniated region. The present study assessed the SS technique using Leader Line, a strong, low-stretch, and affordable monofilament suture as an alternative to synthetic mesh in perineal hernia repair. Postoperative infections associated with synthetic mesh can be difficult to treat [30]. Leader Line offers improved tissue compatibility, stronger anchorage, and a reduced risk of postoperative infection. The technique relies on the external anal sphincter, which is more flexible and places less stress on the anal canal compared to the TT method. These factors likely contributed to improved outcomes, particularly in cases of bilateral perineal hernia.

Various herniorrhaphy techniques have been developed to address ventromedial weakness in hernia repairs, including the semitendinosus muscle transposition [31], combined transposition of the internal obturator and superficial gluteal muscles [32], and bilateral superficial gluteal muscle flaps [33]. Studies have shown encouraging results for each procedure. While these techniques show promise, the SS procedure offers an alternative method for correcting the ventromedial region. The A-shaped leader line might be particularly advantageous if there is a risk of the hernial sac closing accidentally, potentially affecting the pudendal nerve, sciatic nerve, and pudendal arteries. The SS technique typically has a relatively short surgical duration, and postoperative rectal palpation and surgical site examination revealed no indications of pain, stricture, or constriction of the rectum. While surgeon experience was not specifically evaluated in this study, it is recognized as a factor that can influence repair success [3], and we expect similar results here. Proper suture placement in the SS technique is crucial for good outcomes, as careful positioning of the drill holes ensures secure closure of the ventromedial hernia aspect, the most common site of recurrence. It should be noted that castration performed in conjunction with perineal herniorrhaphy has been shown to improve postoperative outcomes [34]. In the present study, all male dogs underwent castration.

This study has several limitations. As a retrospective design, it relies on previously collected data, which may be subject to biases, and information may be missing. Furthermore, the research was conducted at a single hospital, which may limit the generalizability of the findings. The sample size, although adequate, may not be sufficiently large to draw broad conclusions. Additionally, the selection of surgical technique was influenced by client preferences, finances, and surgeon expertise, potentially introducing bias. The lack of long-term follow-up may have resulted in missing data on recurrence rates or late-onset complications. The single observed case of surgical site infection, which resolved with treatment without suture removal, highlights the risk of infection with any perineal hernia repair method. Finally, this study compared only three techniques, excluding other methods. Future studies should include larger sample sizes, longer follow-ups, and a broader range of surgical techniques.

### Conclusion

This study demonstrated that both the IOMF and SS techniques were effective in repairing perineal hernias in dogs. The SS method had the shortest surgical time and fewer postoperative complications than the TT and IOMF techniques. It was also more successful than the TT procedure and caused fewer complications. In addition, the SS procedure provided a stronger connection to the external anal sphincter through the use of a Leader line linking the pelvic diaphragm to the fourth sacral vertebra and ischial bone. This technique was particularly beneficial in cases of pelvic muscle atrophy or recurrent hernias where other methods, such as IOMF, had failed. The shorter surgical time and reduced risk of infection associated with the SS technique suggest that it is a practical alternative for perineal hernia repair in veterinary practice.

## List of abbreviations

Co, coccygeus; E, external anal sphincter muscle; IC, ischial arch; IO, internal obturator muscle; IOMF, internal obturator muscle flap; LA, levator ani muscle; LL, leader line; SC, subcutaneous; SL, sacrotuberous ligament; SS, sacroischial sling; TT, traditional technique.

## References

[1] Nakaza P, Silva AR, De Brucker AA, Grangeiro DM, Vicente PC, Dos Santos JC, et al. (2022). Unilateral perineal hernia surgery: case report. Rev Fac Med Vet Zootec.

[2] Mann FA, de Mello Souza CH Perineal hernia. In: Small animal soft tissue surgery. Wiley-Blackwell, Hoboken, USA. 2023; pp. 318–30;.

[3] Tobias KM (2015). Perineal hernias. Aronson LR, editor. Small animal surgical emergencies.

[4] Sprada AG, Huppes RR, Feranti JPS, De Souza FW, Coelho LDP, Moraes PC, et al. (2017). Perineal hernia in dogs: which technique should we use?. Acta Sci Vet.

[5] Gill SS, Barstad RD (2018). A review of the surgical management of perineal hernias in dogs. J Am Anim Hosp Assoc.

[6] Hadžijunuzović-Alagić D, Šehić M, Milošević H, Kolašinac SS, Hadžimusić N (2024). Predispositions to degenerative lumbosacral stenosis in dogs: an investigation of breed, gender, and age factors. Int J Vet Sci.

[7] Machado AVLP, Lugoch G, dos Santos API, Gonçalves MEP, De Oliveira MT, Viela JAP, et al. (2020). Perineal hernia in a bitch. Acta Sci Vet.

[8] Kitessa JD, Terefe KB (2022). Perineal herniorrhaphy along with anal sacculectomy in dog: case report. Int J Vet Sci Res.

[9] Sjollema BE, Van Sluijs FJ (1989). Perineal hernia repair in the dog by transposition of the internal obturator muscle: iI. complications and results in 100 patients. Vet Q.

[10] Hardie EM, Kolata RJ, Earley TD, Rawlings CA, Gorgacz EJ (1983). Evaluation of internal obturator muscle transposition in treatment of perineal hernia in dogs. Vet Surg.

[11] Hashimoto Y, Nakagawa T, Nishimura R (2023). Evaluation of semitendinosus muscle transposition for treatment of perineal hernias in 33 small-breed dogs. Can J Vet Res.

[12] Bongartz A, Carofiglio F, Balligand M, Heimann M, Hamaide A (2005). Use of autogenous fascia lata graft for perineal herniorrhaphy in dogs. Vet Surg.

[13] Heishima T, Asano K, Ishigaki K, Yoshida O, Sakurai N, Terai K, et al. (2022). Perineal herniorrhaphy with pedunculated tunica vaginalis communis in dogs: description of the technique and clinical case series. Front Vet Sci.

[14] Pratummintra K, Chuthatep S, Banlunara W, Kalpravidh M (2013). Perineal hernia repair using an autologous tunica vaginalis communis in nine intact male dogs. J Vet Med Sci.

[15] Elkasapy A, Shokry M, Alakraa A, Khalifa O (2022). Prosthetic polyester-based hybrid mesh for repairing of perineal hernia in dogs. Open Vet J.

[16] Heishima T, Ishigaki K, Seki M, Teshima K, Yoshida O, Iida K (2023). Retrospective analysis of perineal herniorrhaphy with cone-shaped polypropylene mesh in dogs: technique description and outcome. Front Vet Sci.

[17] Sharma A, Chandrakala K, Kumari L, Singh S, Kumar S, Kumar P (2016). Successful surgical management of recurrent perineal hernia using colopexy and cystopexy in a dog. Int J Livest Res.

[18] Cinti F, Rossanese M, Pisani G (2021). A novel technique to incorporate the sacrotuberous ligament in perineal herniorrhaphy in 47 dogs. Vet Surg.

[19] Woldberg IWK (2014). Perineal hernia repair in the dog by a modified technique of transposition of the internal obturator muscle: long-term results in 60 patients. Utrecht University Repository, Utrecht, Netherlands.

[20] Maute A, Koch D, Montavon P (2001). Perineal hernia in dogs: colopexy, vasopexy, cystopexy and castration as elective therapies in 32 dogs. Schweiz Arch Tierheilkd.

[21] Bernardé A, Rochereau P, Matres-Lorenzo L, Brissot H (2018). Surgical findings and clinical outcome after bilateral repair of apparently unilateral perineal hernias in dogs. J Small Anim Pract.

[22] Brissot HN, Dupré GP, Bouvy BM (2004). Use of laparotomy in a staged approach for resolution of bilateral or complicated perineal hernia in 41 dogs. Vet Surg.

[23] Shaughnessy M, Monnet E (2015). Internal obturator muscle transposition for treatment of perineal hernia in dogs: 34 cases (1998–2012). J Am Vet Med Assoc.

[24] García AR, Sirvet NP, Flores ED, Villalba AG, Calvo LJE (2015). Perineal hernia in the dog, a prevalence study of 81 cases. Arch Med Vet.

[25] Niebauer GW, Shibly S, Seltenhammer M, Pirker A, Brandt S (2005). Relaxin of prostatic origin might be linked to perineal hernia formation in dogs. Ann N Y Acad Sci.

[26] Hornsby DJ, Poterski RS, Summerlee AJS (2001). Relaxin expression and binding in the rat prostate. Relaxin 2000: Proceedings of the Third International Conference on Relaxin u0026amp; Related Peptides, 2000, Broome, Australia.

[27] Hayashi AM, Rosner SA, De Assumpção TCA, Stopiglia AJ, Matera JM (2016). Retrospective study (2009–2014): perineal hernias and related comorbidities in bitches. Top Companion Anim Med.

[28] Tobias KM, Crombie K (2022). Perineal hernia repair in dorsal recumbency in 23 dogs: description of technique, complications, and outcome. Vet Surg.

[29] Moreira PDP, Cardoso MRP, Rosado IR, Sampaio RL, Soares FDO, Martin I, et al. (2021). Perineal hernia in dogs. Acta Sci Vet.

[30] Ramos RD, O’Brien WJ, Gupta K, Itani KM (2021). Incidence and risk factors for long-term mesh explantation due to infection in more than 100,000 hernia operation patients. J Am Coll Surg.

[31] Morello E, Martano M, Zabarino S, Piras LA, Nicoli S, Bussadori R, et al. (2015). Modified semitendinosus muscle transposition to repair ventral perineal hernia in 14 dogs. J Small Anim Pract.

[32] Rosselló GC, Turner A, Macías C, Ramírez JM (2017). Combined transposition of internal obturator and superficial gluteal muscles for perineal hernia treatment in dogs: 17 cases (2017-2020). J Small Anim Pract.

[33] Bitton E, Keinan Y, Shipov A, Joseph R, Milgram J (2020). Use of bilateral superficial gluteal muscle flaps for the repair of ventral perineal hernia in dogs: a cadaveric study and short case series. Vet Surg.

[34] Sangmanee P, Kovitvadhi A, Sutthiprapa W, Choochalermporn P, Limmanont C (2025). Canine perineal hernia associated with prostatic disorders: is castration really beneficial? A retrospective study. Animals.

